# Retraction: The Significance of Notch1 Compared with Notch3 in High Metastasis and Poor Overall Survival in Hepatocellular Carcinoma

**DOI:** 10.1371/journal.pone.0301556

**Published:** 2024-04-02

**Authors:** The PLOS ONE Editors

This article [1] was identified as one of a group of articles connected by concerns about image reuse [2, 3, 7–16].

The concerns with this article [1] affect results presented in Figures 2 and 3, as well as cell lines used in this study. Specifically,

- The Figure 2a MHCC97H NT, MHCC97H CS, and MHCC97H N3S panels appear to partially overlap, despite representing different experimental conditions.
- The HepG2 panels in Figures 2a and 2d appear similar to panels published in later publications [2, 3] by different research groups and used to represent different experimental conditions, as follows:
  - Figure 2a HepG2 NT panel of [1], Fig 4 F5M2 panel of [2], and Fig 4C Eca-109/pCMV-SOX2 (migrated) panel of [3].
  - Figure 2a HepG2 CS panel of [1], Fig 4 F5M2,NC panel of [2], and Fig 4C Eca-109/pCMV-SOX2-Ctr siRNA (migrated) panel of [3].
  - Figure 2a HepG2 N1S panel of [1], Fig 4 F5M2,Low 144 panel of [2], and Fig 4C Eca-109/pCMV-SOX2-STAT3 siRNA (migrated) panel of [3].
  - Figure 2a HepG2 N3S panel of [1], Fig 4 F5M2,Inhibitor panel of [2], and Fig 4C Eca-109/pCMV-SOX2-Ctr siRNA (invasive) panel of [3].
  - Figure 2d HepG2 NT panel of [1] and Fig 4C Eca-109/pCMV-SOX2 (invasive) panel of [3].
  - Figure 2d HepG2 CS panel of [1] and Fig 4C Eca-109/pCMV-SOX2-HIF-1a siRNA (migrated) panel of [3].
  - Figure 2d HepG2 N1S panel of [1] and Fig 4C Eca-109/pCMV-SOX2-STAT3 siRNA (invasive) panel of [3].
  - Figure 2d HepG2 N3S panel of [1] and Fig 4C Eca-109/pCMV-SOX2-HIF-1a siRNA (invasive) panel of [3].
- In Figure 3a, there appear to be irregular patterns in the background of the left MMP-9 panel and the right uPA panel.
- Concerns have been raised about the identity of cell lines reported in the study that raise questions about the relevance of the obtained results for hepatocellular carcinoma.
  - HL-7702: This article [1] presents the HL-7702 cell line as a human non-tumor cell line. However, after the publication of this article [1], the parent cell line for this cell line (L-02) was shown to be a contaminated cell line and is a potential derivative of HeLa [4].
  - SMMC-7221: This article [1] presents the SMMC-7221 cell line as a hepatocellular carcinoma cell line. However, after the publication of this article [1], this cell line was shown to be a contaminated cell line and is a potential derivative of HeLa [4, 5].
  - Hep-G2: This article [1] presents the Hep-G2 cell line as a hepatocellular carcinoma cell line. However, prior to the publication of this article [1], this cell line had been shown to be derived from a hepatoblastoma [6].

First author LZ stated that errors were made during the preparation of the Figure 2A MHCC97H results and provided replacement data. However, upon editorial review of these data, additional partial panel overlap was observed.

In light of the unresolved concerns that question the validity and reliability of the reported data, the *PLOS ONE* Editors retract this article.

LZ and KD disagreed with the retraction. NZ, WSong, NY, QL, WSun, YZ, and DW either did not respond directly or could not be reached.
